# A pediatric regimen for adolescents and young adults with Philadelphia chromosome‐negative acute lymphoblastic leukemia: Results of the ALLRE08 PETHEMA trial

**DOI:** 10.1002/cam4.2814

**Published:** 2020-02-05

**Authors:** Josep‐Maria Ribera, Mireia Morgades, Pau Montesinos, Mar Tormo, Daniel Martínez‐Carballeira, José González‐Campos, Cristina Gil, Pere Barba, Raimundo García‐Boyero, Rosa Coll, María Pedreño, Jordi Ribera, Santiago Mercadal, Susana Vives, Andrés Novo, Eulàlia Genescà, Jesús‐María Hernández‐Rivas, Juan Bergua, María‐Luz Amigo, Ferran Vall‐Llovera, Pilar Martínez‐Sánchez, María Calbacho, Irene García‐Cadenas, Antoni Garcia‐Guiñon, María‐José Sánchez‐Sánchez, Marta Cervera, Evarist Feliu, Alberto Orfao, the PETHEMA Group, Spanish Society of Hematology

**Affiliations:** ^1^ Departments of Clinical Hematology ICO‐Hospital Germans Trias i Pujol. Josep Carreras Research Institute Universitat Autònoma de Barcelona Barcelona Spain; ^2^ Departments of Clinical Hematology Hospital Universitari i Politècnic La Fe CIBERONC, Instituto Carlos III Valencia Spain Madrid Spain; ^3^ Departments of Clinical Hematology Hospital Clínico Valencia Valencia Spain; ^4^ Hospital Central de Asturias Oviedo Spain; ^5^ Departments of Clinical Hematology Hospital Universitario Virgen del Rocío Sevilla Spain; ^6^ Departments of Clinical Hematology Hospital General Universitario de Alicante Alicante Spain; ^7^ Departments of Clinical Hematology Hospital Universitari Vall d’Hebron Universitat Autònoma de Barcelona Barcelona Spain; ^8^ Departments of Clinical Hematology Hospital General Universitario de Castellón Castelló Spain; ^9^ Departments of Clinical Hematology ICO‐Hospital Josep Trueta Girona Spain; ^10^ Departments of Clinical Hematology Hospital Dr. Peset Valencia Spain; ^11^ Departments of Clinical Hematology ICO‐Hospital Duran i Reynals, L’Hospitalet de Llobregat Barcelona Spain; ^12^ Departments of Clinical Hematology, Hospital Son Espases Palma de Mallorca Spain; ^13^ Departments of Clinical Hematology Hospital Clínico Universitario, Cancer Research Center (IBMCC‐CSIC/USAL), Cytometry Service CIBERONC Salamanca Spain; ^14^ Departments of Clinical Hematology Hospital San Pedro de Alcántara Cáceres Spain; ^15^ Departments of Clinical Hematology Hospital Morales Meseguer Murcia Spain; ^16^ Departments of Clinical Hematology Hospital Mútua de Terrassa Terrassa Spain; ^17^ Departments of Clinical Hematology Hospital Doce de Octubre Madrid Spain; ^18^ Departments of Clinical Hematology Hospital Ramón y Cajal Madrid Spain; ^19^ Departments of Clinical Hematology Hospital de Sant Pau Barcelona Spain; ^20^ Departments of Clinical Hematology Hospital Arnau de Vilanova Lleida Spain; ^21^ Departments of Clinical Hematology Hospital Lucus Augusti Lugo Spain; ^22^ Departments of Clinical Hematology ICO‐Hospital Joan XXIII Tarragona Spain

**Keywords:** acute lymphoblastic leukemia, adolescents and young adults, pediatric treatment

## Abstract

**Background:**

Pediatric‐based or ‐inspired trials have improved the prognosis of adolescents and young adults (AYA) with Philadelphia chromosome‐negative (Ph‐neg) acute lymphoblastic leukemia (ALL).

**Methods:**

This study reports the results of treatment of the ALLRE08 trial, a full pediatric trial for AYA aged 15‐30 years with standard‐risk (SR) ALL.

**Results:**

From 2008 to 2018, 89 patients (38 adolescents [15‐18 years] and 51 young adults [YA, 19‐30 years], median age: 20 [15‐29] years) were enrolled in the ALLRE08 trial. The complete response (CR) was 95%. Twenty‐two patients were transferred to a high‐risk (HR) protocol because of poor marrow response on day 14 (*n* = 20) or high‐level of end‐induction minimal residual response (MRD ≥ 0.25%, *n* = 2). Cumulative incidence of relapse (CIR) at 5 years was 35% (95%CI: 23%‐47%), with significant differences between adolescents and YA: 13% (4%‐28%) vs 52% (34%‐67%), *P* = .012. No treatment‐related mortality was observed in 66/66 patients following the ALLRE08 trial vs 3/23 patients moved to a HR trial. The estimated 5‐year overall survival (OS) was 74% (95%CI: 63%‐85%), with significantly higher rates for adolescents vs YA: 87% (95%CI: 74%‐100%) vs 63% (46%‐80%), *P* = .021. Although CIR or OS were lower in patients who were transferred to a HR trial, the differences were not statistically significant (CIR: 34% [21%‐47%] vs 37% [14%‐61%]; OS: 78% [66%‐90%] vs 61% [31%;91%]).

**Conclusion:**

A full pediatric trial is feasible and effective for AYA with Ph‐neg, SR‐ALL, with better results for adolescents than for YA. Outcome of patients with poor early response rescued with a HR trial was not significantly inferior.

## Graphical abstract

A full pediatric trial is feasible and effective for adolescent and young adults with acute lymphoblastic leukemia, with better results for adolescents than for young adults. The outcome of patients showing poor early response was not significantly inferior than that observed for good responders after being transferred to a high‐risk trial.

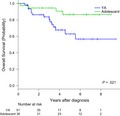

## 1 INTRODUCTION

In recent years, the results of treatment of adolescents and young adults (AYA) diagnosed with ALL have significantly improved with the use of pediatric‐based or ‐inspired protocols.^1^, ^2^, ^3^ This was first observed when retrospective studies demonstrated clear differences in outcomes for AYA depending on enrollment in pediatric vs adult cooperative group studies, despite most adult treatment regimens evolving from a pediatric background. These improvements have also been confirmed in population‐based studies.^4^ However, the design of pediatric trials significantly differs from that of adult trials.^5^ Pediatric trials involve more intensive dosing of key therapeutic agents in ALL, including vincristine, steroids, and asparaginase, while fewer myelosuppressive drugs such as anthracyclines and cytarabine are used in these trials. In addition, hematopoietic stem cell transplantation (HSCT) is less used in pediatric trials.^6^, ^7^ Other factors contributing to the differences between the two types of trials relate to the greater experience observed in pediatric centers and higher adherence of AYA treated in pediatric vs adult cancer centers in some countries.^7^, ^8^, ^9^, ^10^ However, a recently published study by three adult US cooperative groups (CALGB, ECOG, and SWOG) demonstrated that the use of a full pediatric regimen for young adults (YA) with ALL up to the age of 40 years was feasible and effective, resulting in improved survival rates compared to historical controls.^11^

In 1996 the Spanish PETHEMA (*Programa Español de Tratamientos en Hematología*) Group conducted a full pediatric trial (ALL96) to treat Philadelphia chromosome‐negative (Ph‐neg) AYA up to the age of 30 years, showing no significant differences in outcome between adolescents (15‐18 years) and YA (19‐30 years).^12^ At the time of the design of the trial the assessment of minimal residual disease (MRD) for tailored treatment was not extensively employed. In 2007, the PETHEMA Group developed another trial (ALLRE08) incorporating MRD to the decision‐making process without changing the inclusion criteria and the chemotherapy schedule. This study reports the outcomes of the Ph‐neg AYA ALL patients included in this latter trial.

## 2 PATIENTS AND METHODS

### 2.1 Eligibility

From August 2008 to April 2018, adolescents (age range: 15 to 18 years) and YA age range: 19 to 30 years) with standard‐risk (SR) Ph‐neg ALL were enrolled in the ALLRE08 PETHEMA trial (clinicaltrials.gov identifier: NCT02036489). SR‐ALL was defined for both B‐cell precursor ALL or T‐ALL patients who fulfilled all the following criteria: a white blood cell count ≤ 30×10^9^/L, absence of t(9;22)/*BCR‐ABL* rearrangements or t(4;11), or any other 11q23/*KMT2A* gene rearrangement. This definition base on a minimal set of criteria was identical to that of the ALL‐96 trial and allowed a homogeneous recruitment of patients by the participating centers. Patients were not eligible if they had previously received antileukemic treatment, or had uncontrolled or severe cardiovascular (history of congestive heart failure [New York Heart Association class III or IV] or left ventricular ejection < 40%), hepatic (total serum bilirubin > 1.5 × the upper normal limit [ULN], not attributable to ALL) or renal disease (serum creatinine > 1.5 × ULN and estimated creatinine clearance < 40 mL/minute [Cockcroft‐Gault formula] not attributable to ALL) or had a severe psychiatric condition (mental illness that does not allow to follow the protocol according to the physicians criteria). Patients with mature B ALL, Ph‐positive ALL, T or B‐cell lymphoblastic lymphoma or those with mixed phenotype acute leukemia were not included in the trial. Patients were centrally registered after informed consent was obtained from adult patients or from parents or guardians for patients under 18 years. The study was approved by the Institutional Review Boards of all the participating centers.

### 2.2 Diagnostic procedure

ALL was defined based on bone marrow (BM) infiltration of > 20% of blasts with lymphoid morphology. Immunophenotyping was performed by flow cytometry with monoclonal antibodies against with B‐cell, T‐cell, myeloid, and precursor cell‐associated antigens, and B or T lineage commitment was defined based on the WHO 2008 criteria. Chromosomal analyses (using direct methods and unstimulated short‐term cultures with G‐banding) of BM or peripheral blood (PB) samples performed at diagnosis were centrally reviewed. A minimum of 20 metaphase cells were required to define a normal karyotype. Fluorescence in situ hybridization (FISH) studies for *BCR‐ABL1*, *KMT2A*, *MYC,* and *TCF3‐PBX1* were performed in cases without a valid cytogenetic study.

### 2.3 Minimal residual disease assessment

Bone marrow MRD levels were assessed at each participating center in CR patients at: i) end of induction (weeks 5‐6) in CR patients, ii) end of consolidation/reinduction (weeks 19‐20), iii) end of the fourth reinduction cycle (weeks 25‐26), and iv) end of maintenance (week 106‐108) using a 4‐color multiparameter flow cytometry (FCM) approach with a limit of detection of 10^‐4^.

### 2.4 Treatment and response criteria

Treatment (Table 1) consisted of a prephase with prednisone (maximum of 1 week) and one dose of triple intrathecal therapy (TIT) with methotrexate, cytarabine, and hydrocortisone given while ALL was being fully characterized. Subsequently, the patients received induction therapy. Patients with morphologic CR and lower MRD levels (defined as < 0.25% in this trial) received consolidation therapy followed by reinduction. Maintenance therapy with monthly reinforcement cycles (M‐1) was administered up to 1 year after ALL diagnosis. Then a second phase of maintenance therapy without reinforcement (M‐2) with mercaptopurine plus methotrexate was administered up to 2 years to patients in continuous CR. CNS prophylaxis included the administration of 14 courses of TIT over the 2 years of therapy. Cumulative doses of the main cytotoxic drugs were as follows: 24 mg vincristine, 5,150 mg/m^2^ prednisone, 175 mg/m^2^ dexamethasone, 320,000 IU/m^2^ native *E coli* asparaginase (pegylated formulation of asparaginase was not licensed in Spain at the time of this protocol), 240 mg/m^2^ daunorubicin, 2,200 mg/m^2^ cyclophosphamide, and 9,000 mg/m^2^, 24‐h continuous IV infusion methotrexate plus 1,560 mg/m^2^ IM methotrexate during maintenance. Hospitalization, prophylaxis and management of infections, transfusion, and other supportive care therapies were carried out according to institutional protocols. Therapeutic dose monitoring of asparaginase was not performed.

**Table 1:** PETHEMA ALLRE08 protocol. Chemotherapy schedule

Phase Drugs	Week	Route	Dose	Days
**Prephase** a				
Prednisone	1	IV	60 mg/m2	1‐7
Triple IT therapy	1	IT		1
MTX	1	IT	15mg	1
ARA‐C	1	IT	30 mg	1
Hydrocortisone	1	IT	20 mg	1
**Remission induction**				
Vincristine	1‐4	IV	2 mg (absolute)	1, 8, 15, 22
Prednisone	1‐5	IV/PO	60 mg/m2	1‐27
		IV/PO	30 mg/m2	28‐35
Asparaginase (*E coli*, native)	2‐4	IV	10,000 IU/m2	1‐12, 17‐19, 24‐26
	5	IV	1,000 mg/m2	35
Cyclophosphamide	1, 5	IT		1, 29
Triple IT Therapy				
**Consolidation**				
Mercaptopurine	1	PO	50 mg/m2	1‐7
Methotrexate	1, 4, 8	IV (24h)	3 g/m2 b	1, 28, 56
Teniposide			150 mg/m2/12h	14, 42
ARA‐C	2,6	IV		14, 42
Triple IT therapy	2,6 1, 4, 8	IV IT	500 mg/m2/12h	1, 28, 56
**Reinduction**				
Dexamethasone	1, 2	IV/PO	10 mg/m2	1‐14
Vincristine	1‐3	IV/PO IV	5 mg/m2 2 mg (absolute)	15‐21 1, 8, 15
Daunorubicin	1, 2	IV	30 mg/m2	1‐2, 8‐9
Asparaginase (*E coli*, native)	1, 3	IM/IV	10,000 IU/m2	1‐3, 15‐17
Triple IT Therapy	1, 3	IT		1, 15
**Maintenance with reinforcement**				
Maintenance		IM	20 mg/m2/week	Until week 52
Methotrexate		PO		Until week 52
Mercaptopurine			50 mg/m2/d	
Reinductions	1	IV		1, until week 52
Vincristine	1	IV/PO	2 mg (absolute)	1‐7, until week 52
Prednisone	1	IV	60 mg/m2	1, until week 52
Asparaginase (*E coli*, native)			20,000 IU/m2	
**Maintenance without reinforcement**				
Methotrexate		IM	20 mg/m2/week	Until week 105
Mercaptopurine		PO	50 mg/m2/d	Until week 105

Abbreviations: ARA‐C, cytarabine; IM, intramuscular; IT, intrathecal; IV, intravenous; MTX, methotrexate; PO, oral.

a Duration of less than 1 week if ALL has been well characterized.

b With folinic acid rescue.

Patients with poor early cytological response (defined as BM blast cells ≥ 10% on day 14 of induction), or MRD level ≥ 0.05% at the end of reinduction or after the 4th monthly reinforcement cycle were considered as high‐risk (HR) patients and entered in the ALL‐HR PETHEMA trial active at that time (ALLHR03^13^ [NCT 00853008] until 2010 and ALLHR11^14^ [NCT01540812] from 2011 to 2019) (Table S1).

### 2.5 Outcome assessment

The primary endpoints of this study were: i) CR rate, ii) cumulative incidence of relapse (CIR), and iii) overall survival (OS). Secondary objectives included assessment of toxicity and comparison with ALL96 trial outcomes (updated for this study). Planned enrollment was of 90 patients to allow comparison with the ALL96 trial.

CR was defined as the absence of clinical manifestations of ALL, neutrophil count > 1.5×10^9^/L, platelet count > 150×10^9^/L, and hemoglobin level > 100 g/L, with < 5% of blast cells in BM. Patients with ≥ 5% BM blast cells at the end of the induction phase were considered as induction failures. Two patterns of early response were considered in BM aspirate performed on day 14 of treatment: 1) slow, defined as the presence of ≥ 10% of blast cells, and 2) standard, defined as < 10% BM blast cells or hypoplastic BM. Overall survival was defined as the time from study entry to death or last follow‐up. Event‐free survival was defined as the time from diagnosis to failure, relapse or death by any cause or last follow‐up. The Common Terminology Criteria for Adverse Events (CTCAE v 3.0) was used for analysis of toxicity.

### 2.6 Statistical analysis

Patients’ follow‐up was updated on September 2018. The median test was used to compare quantitative variables, while the Chi‐square or Fisher's exact tests were employed to assess differences in proportions. All comparisons were two‐tailed. Curves for OS and event‐free survival (EFS) were plotted according to the Kaplan‐Meier method and were compared by the log‐rank test. The CIR was calculated using cumulative incidence functions by competing risks analysis, nonrelapse mortality (NRM) being the competing event. Data collection and statistical analyses were performed at the PETHEMA Data Center for ALL using SPSS v.24 and R v.3.5.2 software.

## 3 RESULTS

### 3.1 Patients features at diagnosis

One‐hundred and two patients were enrolled in 40 Spanish hospitals, 89 of whom were eligible for the study. The remaining 13 patients were excluded because age < 15 years (*n* = 1), ALL with high‐risk features at baseline (*n* = 9), lymphoblastic lymphoma (*n* = 2), and Philadelphia chromosome‐positive ALL (*n* = 1).

Table 2 shows the main clinical and biological features of the whole series including adolescents (*n* = 38) vs YA (*n* = 51). Median age at diagnosis was 20 years (range 15‐30 years) and 54 patients (61%) were males. Extramedullary disease was present at diagnosis in four cases (5%). Eighty‐four patients (94%) had B‐cell precursor ALL. Twenty‐three patients (26%) showed a normal karyotype. Abnormal karyotypes included high hyperdiploidy (*n* = 8, 9%), t(1;19) (*n* = 4, 5%), t(12;21)(*ETV6/RUNX1*) (*n* = 1, 1%) and del(9p) (*n* = 3, 3%), among others. Four cases (5%) had a complex karyotype, an abnormality not considered as HR at the time of protocol design. The absence or insufficient number of metaphases was registered in 28 (32%) cases. No significant differences were observed in karyotypic results on comparison between adolescents and YA (Table 2).

**Table 2:** Patient characteristics in the whole series and according to the age group

Characteristic	Whole series (*n* = 89)	Adolescents (*n* = 38)	Young adults (*n* = 51)	*P* value
Age at diagnosis, years, median (range)	20 (15‐29)	17 (15‐18)	23 (19‐29)	<0.001
Gender				0.197
Male	54 (61%)	26 (68%)	28 (55%)	
Female	35 (39%)	12 (32%)	23 (45%)	
Performance status (ECOG scale)				0.746
0	26 (30%)	13 (34%)	13/48 (27%)	
1	52 (61%)	22 (58%)	30/48 (63%)	
2	7 (8%)	3 (8%)	4/48 (8%)	
3	1 (1%)	0	1/48 (2%)	
Extramedullary disease				NA
CNS	3 (3%)	0	3 (3%)	
CNS + other	1 (1%)	0	1 (1%)	
WBC count, x10^9^/L, median (range)	6.83 (0.4‐30)	6.82 (0.4‐24.8)	7.19 (0.8‐30)	0.736
Phenotype				0.884
Early pre‐B	0	0	0	
Common	68 (76%)	30 (79%)	38 (74%)	
Pre‐B	16 (18%)	6 (16%)	10 (20%)	
T	5 (6%)	2 (5%)	3 (6%)	
Karyotype				0.452
No growth Normal	28 (32%) 23 (26%)	9 (24%) 10 (26%)	19 (37%) 13 (25%)	
Hyperdiploidy > 50 chr	8 (9%)	5 (13%)	3 (6%)	
t(12;21)/TEL‐AML1	1 (1%)	1 (3%)	0	
t(1;19)	4 (5%)	2 (5%)	2 (4%)	
Other abnormalities	25	11 (29%)	14 (28%)	

Abbreviations: chr, chromosomes; CNS, central nervous system; ECOG, Eastern Cooperative Oncology Group; NA, not available; WBC, white blood cell.

### 3.2 Treatment outcome

**Figure 1:**
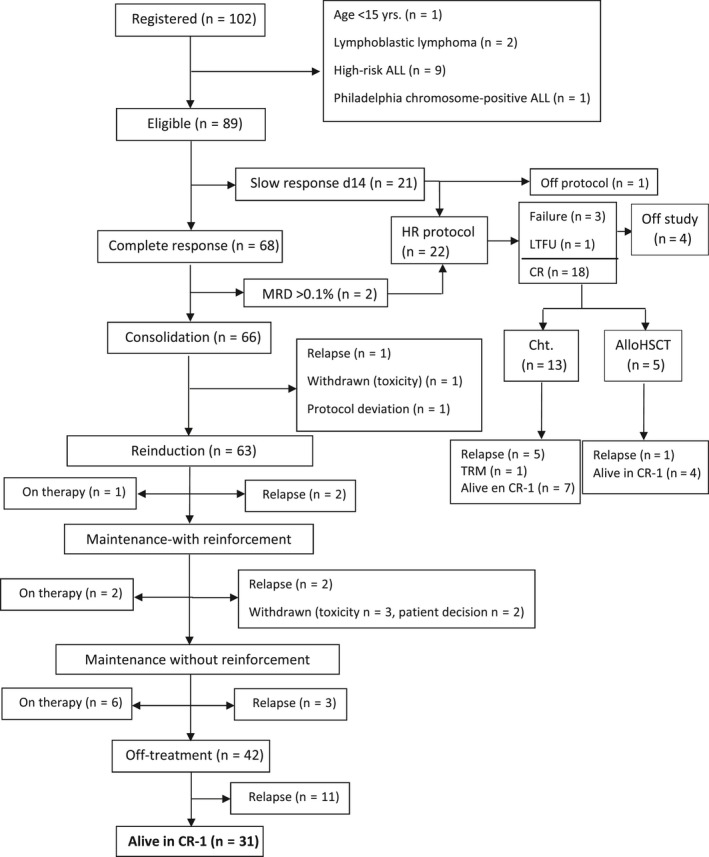
Flow chart of the patients in the ALLRE08 PETHEMA trial

There were no deaths in induction. On day 14 of induction therapy 68 patients (76%) showed standard BM response while 21 patients (24%) showed slow response, and 20 of these latter patients were moved to a HR protocol; the remaining patient withdrew the study. All patients with standard early response achieved CR, and 16 of the remaining 20 patients with slow response achieved CR with the induction therapy of the HR protocol (three patients were resistant and one was not evaluable due to withdrawal from the protocol during induction), for a total of 84 CR patients (95%) (Figure 1). Two CR patients with standard early cytologic response showed high MRD levels (0.8% and 0.75%, respectively) at the end of induction and were then moved to a HR‐ALL protocol. Thus, a total of 66 patients continued with the ALL RE08 trial, and 22 (10 adolescents and 12 YA) were transferred to a HR‐ALL trial (7 to ALLHR03 and 15 to ALLHR11) (Figure 1).

At the time of the analysis 9/66 patients who followed the ALLRE08 trial were receiving consolidation or maintenance therapy at the time of the analysis, 8 patients relapsed during consolidation (*n* = 3) or maintenance (*n* = 5) therapy and 11 patients relapsed off therapy. Seven patients abandoned the protocol due to excess toxicity (*n* = 4), their own decision (*n* = 2) or a major deviation from the protocol (*n* = 1). No therapy‐related deaths were registered. Regarding those 18 patients in CR after inclusion in the HR protocol, 13 received chemotherapy and 5 allogeneic HSCT. Six patients relapsed (5 during chemotherapy and 1 after HSCT), 1 died by toxicity of chemotherapy, and 11 are in CR (7 after chemotherapy and 4 after HSCT). Fourteen patients remain alive (11 in first CR and 3 in second CR).

**Figure 2:**
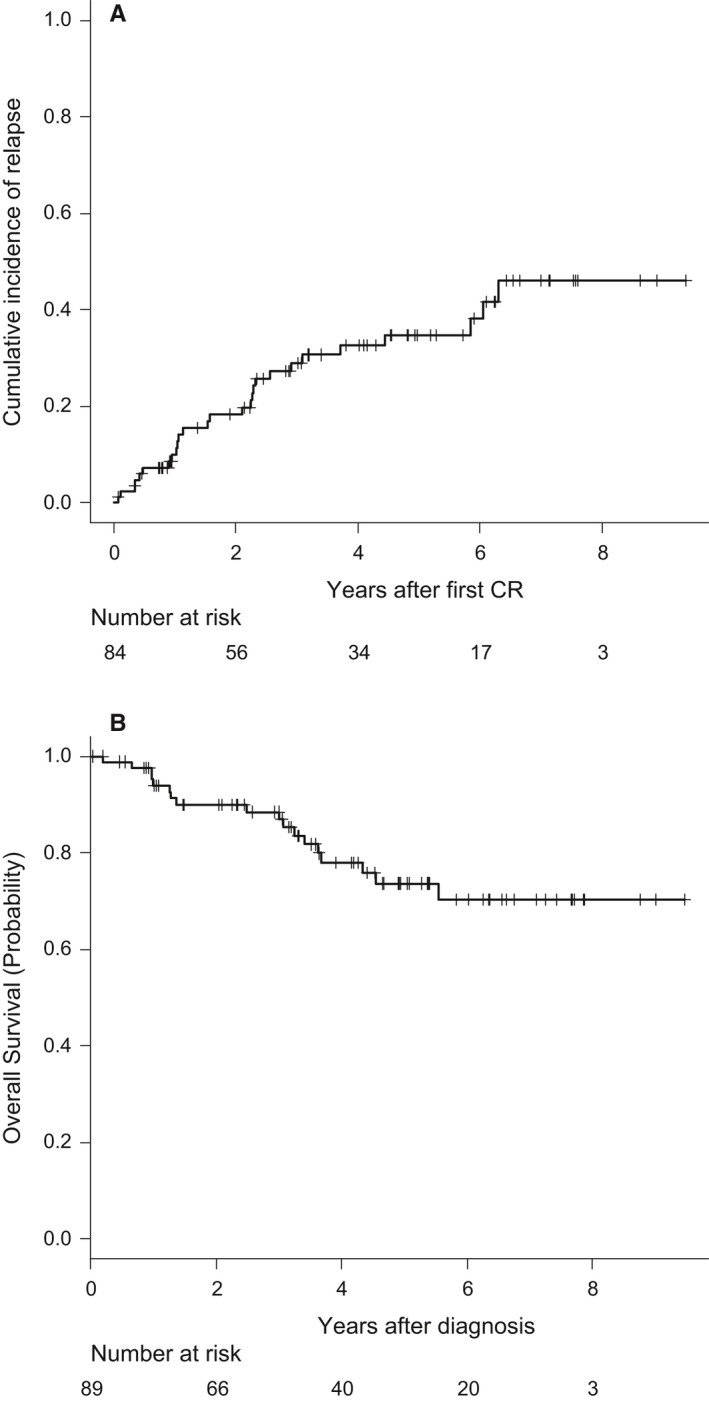
Cumulative incidence of relapse (panel A), and overall survival (panel B) in the whole series

**Figure 3:**
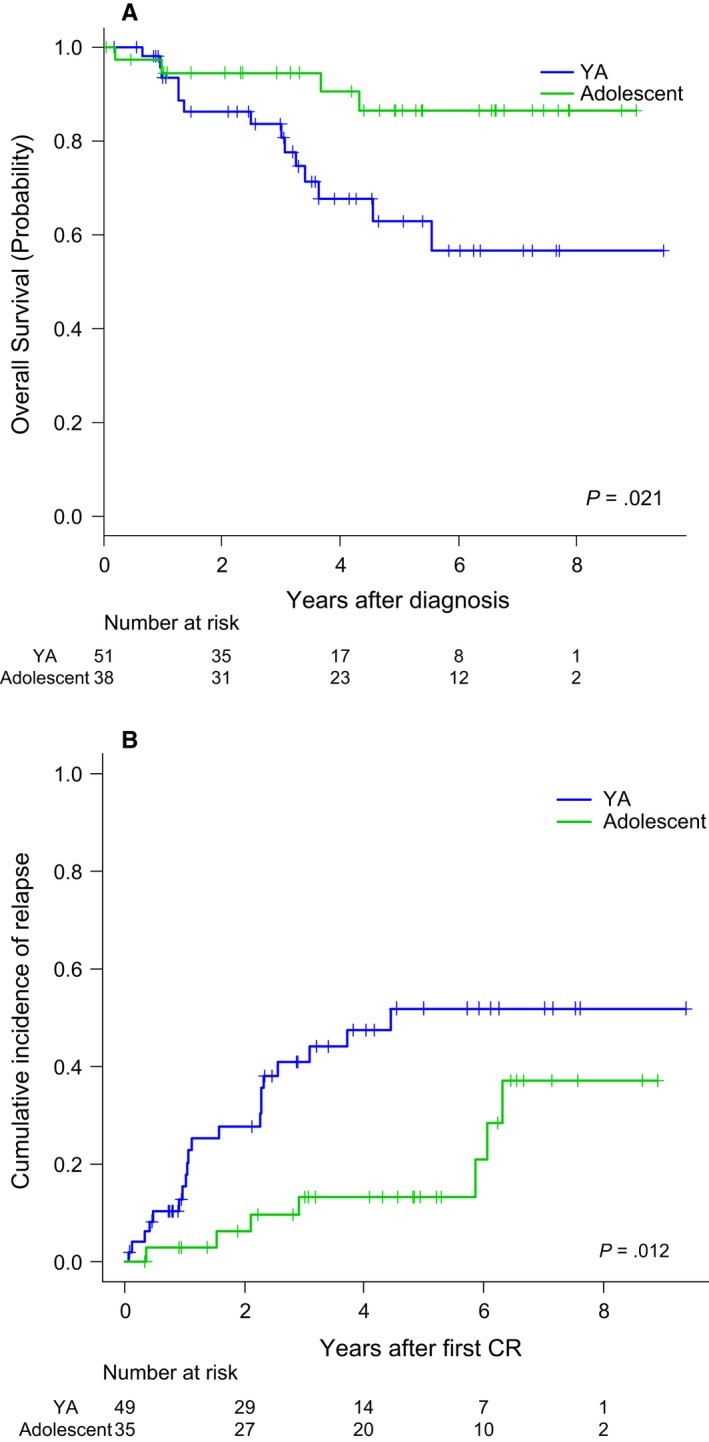
Comparison between the overall survival (panel A), and the cumulative incidence of relapse (panel B) between adolescents and young adults

**Figure 4:**
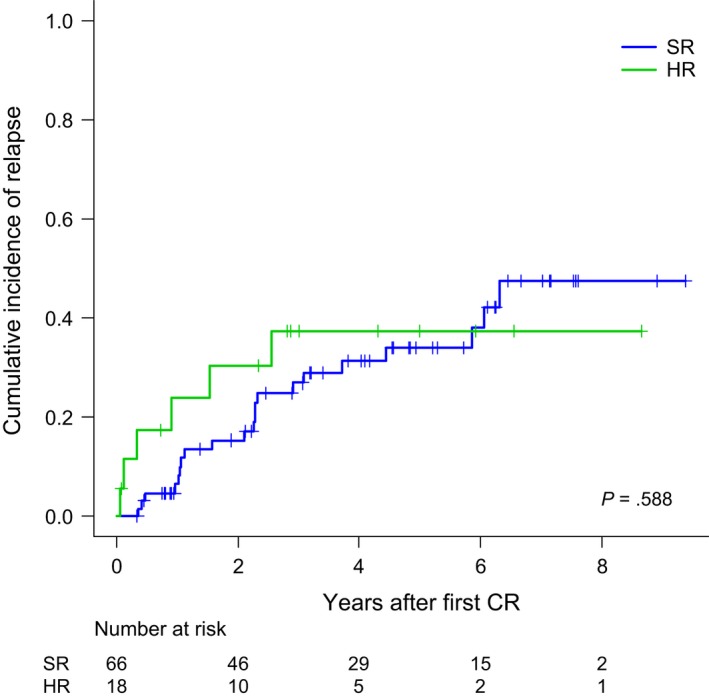
Cumulative incidence of relapse of patients who remained in the ALLRE08 protocol vs those who were moved to a high‐risk (HR) protocol

Overall, 25 patients relapsed in either BM (*n* = 19) or BM plus CNS (*n* = 2), BM and testicle (*n* = 1) BM and lymph nodes (*n* = 1), CNS (*n* = 2) and testicular (*n* = 1). Ten patients were alive in second CR, one was alive in relapse, seven dead by treatment‐related mortality (either by alloHSCT [*n* = 2] or by rescue chemotherapy [*n* = 5]) and seven dead by disease progression. The CIR at 5 years for the whole series was 35% (95%CI: 23%‐47%) (Figure 2A). With a median follow‐up of 4.19 years (range 0.04‐9.47), 18 patients have died, with the OS probability at 5 years being 74% (95%CI: 63%‐85%) for the whole series (Figure 2B). The main causes of death were disease progression (*n* = 15), transplant‐related (*n* = 2) and toxicity of HR chemotherapy (*n* = 1). Fifty‐one patients remained alive and off therapy (41 in first CR and 10 in second CR). Figure 2C shows the EFS for the whole series (probability at 5 years 62% [95%CI: 49%‐73%]). Table 3 shows the main outcomes for both the whole series and for those patients who remained in the ALLRE08 protocol, those who were moved to a HR protocol, and for adolescents and YA considered separately. As shown, significant differences in OS and CIR were observed in adolescents vs YA, with 5 years. OS rates of 87% (95%CI: 74%‐100%) vs 63% (46%‐80%), respectively, *P* = 0.021 and CIR rates of 13% (4%‐28%) vs 52% (34%‐67%), *P* = 0.012, respectively (Figures 3A,B). Interestingly, no significant differences were observed in the CIR in patients who remained in the ALLRE08 protocol vs those who were moved to a HR protocol (5‐year CIR rate of 34% [21%‐47%] vs 37% [14%‐61%], respectively, *P* = 0.588) (Figure 4).

**Table 3:** Main outcomes of the patients from the whole series, for those who remained in the ALLRE08 protocol and those who were moved to high‐risk protocol, and for adolescents and young adults separately

Patient group	N	OS (5‐yrs, 95% CI)	EFS (5‐yrs, 95% CI)	CIR (5‐yrs, 95% CI)
Whole series	89	74% (63%;85%)	62% (49%;73%)	35% (23%;47%)
Patients that remained in the ALLRE08 trial	66	78%^a^ (66%;90%)	67%^e^ (51%;78%)	34%a^b^ (21%;47%)
Patients moved to HR trials	22	61%^a^ (31%;91%)	48%^e^ (25%;68%)	37%^b^ (14%;61%)
Adolescents	38	87%^c^ (74%;100%)	78%^f^ (59%,;89%)	13%^d^ (4%;28%)
Young adults	51	63%^c^ (46%;80%)	49%^f^ (31%;65%)	52%^d^ (34%;67%)

Abbreviations: CIR, cumulative incidence of relapse; EFS, event‐free survival; HR, high risk; OS, overall survival.

a p = 0.343

b p = 0.588

c p = 0.021

d p = 0.012

e p = 0.028

f p = 0.151

### 3.3 MRD status

MRD at the end of the induction was evaluable in 61/68 patients with standard response. Of these, 53 showed MRD level < 0.1%, and 8 had MRD levels ≥ 0.1% (≥0.5% in two, who were moved to a HR protocol). Forty‐eight patients showed end‐induction MRD levels < 0.05%. MRD levels postconsolidation were evaluable in 54/66 patients who began consolidation. The reasons for lack of MRD data in the remaining 12 patients were: early relapse (*n* = 3), withdrawal from protocol (*n* = 1), protocol deviation (*n* = 1), still on therapy (*n* = 1), and no test performed (*n* = 6). No patient was moved to a HR protocol at this time point, because the MRD level remained < 0.05% in all evaluable patients. From those 60 patients who began the maintenance therapy 47 had MRD assessed, while 13 did not because of relapse (*n* = 1), withdrawal from protocol (*n* = 5), still on therapy (*n* = 1) and MRD testing not performed (*n* = 6). Again, no patient was transferred to a HR protocol due to MRD levels < 0.05%. MRD testing at end‐of‐therapy was performed in 27 patients. The remaining 24 patients had no MRD data because they had relapsed (*n* = 3), have just finished M‐2 therapy (*n* = 6) or had missing data (*n* = 15). In 6/11 patients who relapsed off therapy MRD levels at the end of maintenance were < 0.05% while were missing in the remaining five cases.

### 3.4 Dose modifications and treatment toxicity

The dose of chemotherapy was delayed in 1/68 (1.5%) evaluable patients during induction, in 22/62 (35%) evaluable patients in C1, 10/55 (18%) evaluable patients in C2 and in 13/52 (25%) evaluable patients during maintenance. In addition, there were dose modifications of cytotoxic drugs in 2/68 (3%) evaluable patients during induction, in 8/62 (13%) evaluable patients during C1, in 9/55 (16%) evaluable patients during C2, and in 34/52 (65%) patients during maintenance. No differences were found in the frequency of delays or dose modifications of the chemotherapy between adolescents and YA. Of note, OS and CIR were not significantly different in patients who showed delays or dose modifications vs those who received the schedule timely and at full dose (5‐year OS: 76% [62%‐90%] vs 84% [64%‐100%], *P* = 0.326; 5‐year CIR: 32% [14%‐46%] vs 27% [0%‐47%], *P* = 0.920). Main grade III‐IV toxicities observed in the trial (Table 4) were: i) hematologic, particularly in induction, C1 and C2. Despite this relatively high frequency, the number of severe infections was low, ii) hepatic, and iii) hypersensitivity to native *E coli* asparaginase. As expected, the latter was more frequent after its reexposure in C2 cycle or in M1. *Erwinia* asparaginase was given to 9 of 13 patients with grade III‐IV allergic reactions, and removal of asparaginase was done in the remaining four cases.

**Table 4:** Main grade III‐IV toxicities observed per treatment phases of the ALLRE08 trial

	Induction	Consolidation	Reinduction	Maintenance with reinforcements
Neutropenia Days, median [min;max]	55/67 (82%) 15 [1; 41]	34/58 (59%) 6 [1; 30]	26/56 (46%) 3,50 [1 ;13]	5/52 (10%)
Thrombocytopenia Days, median [min;max]	26/65 (40%) 8 [1; 35]	8/58 (14%) 3 [1; 13]	1/55 (2%) 3	0
Coagulation disorders	9/67 (13%)	1/59 (2%)	1/56 (2%)	0
Infection	21/65 (32%)	14/59 (24%)	5/56 (9%)	1/52 (2%)
Hypersensitivity	0	1/59 (2%)	8/56 (14%)	4/52 (8%)
Neurologic	2/68 (3%)	0	2/56 (4%)	1/52 (2%)
Hepatic	13/68 (19%)	8/58 (14%)	5/56 (9%)	6/52(12%)
Renal	0	2/59 (3%)	0	0
Gastrointestinal	0	0	1/56 (2%)	0
Vascular	2/67 (3%)	0	1/56 (2%)	0
Pulmonary	0	0	1/56 (2%)	1/52 (2%)
Metabolic	2/67 (3%)	0	0	0

### 3.5 Patients outcome in the ALLRE08 vs the ALL‐96 PETHEMA trials

Table S1 shows the comparison of the main outcomes between these protocols. As expected, the outcomes were not significantly different, although a significantly lower OS was observed in YA vs adolescents in the current study and not in the ALL96 trial.

## 4 DISCUSSION

This study shows that treatment with a full pediatric protocol is feasible and effective for AYA with Ph‐neg, SR‐ALL, with better results for adolescents than for YA. Of note, one third of the patients showed poor early response, but their outcome was not significantly inferior than that observed for the good responders after being rescued with a HR therapy.

Adolescent and YA patients with ALL represent a population with specific characteristics and needs.^1^, ^2^, ^3^ At present it is well established that adolescents aged 15 to 20 years are best treated with full pediatric protocols; in parallel, growing evidence suggests that this might also be true for YA (with an upper age limit of 30 to 40 years in most studies or even up to 50‐55 years in some studies),^15^, ^16^, ^17^, ^18^ with 5‐year survival rates around 70%, despite HSCT is less used in pediatric trials. Since MRD has emerged as a powerful prognostic marker useful for risk stratification in ALL,^19^ the PETHEMA ALLRE08 trial added MRD assessment to the early cytologic response to select among AYA patients with SR‐ALL those who were poor responders and transfer them to a HR protocol, while maintaining the full pediatric chemotherapy schedule for good responders.

Overall, the CR rate observed was high and similar to that reported for most full pediatric or pediatric‐inspired protocols focused on AYA with ALL.^11^, ^16^, ^17^, ^18^, ^19^, ^20^, ^21^ and those without a pediatric basis.^21^ Of note, one third of patients were poor responders and were transferred per protocol to a HR trial, which allowed CR to be attained in 80% of patients. No toxic toxicity‐related deaths occurred among AYA who followed the ALLRE08 trial and the overall adherence to the protocol being good. Despite this a few patients abandoned the trial due to excess toxicity, their own decision or a major deviation from the predefined therapeutic schedule. In contrast, a significant rate of relapses off therapy (11/19) occurred despite the MRD levels were low in all tested cases. Because no regular MRD assessment was performed off therapy, early reemergence of MRD could not be identified and consequently early therapeutic intervention at an MRD positive CR status was not feasible in these patients. Whether or not prolonged maintenance therapy for 2.5 or 3 years from CR, as done in some pediatric protocols, would result in a lower frequency of late relapses among these patients remains to be investigated.

Among all treated patients those with poor early response were more difficult to treat, showing a higher rate of resistant leukemia and deaths by toxicity. It might well be that a more extensive work‐up of ALL at diagnosis would allow identification of patients with poor genetic risk (eg, *BCR‐ABL1*‐like ALL) among this group of patients.^22^ However, still 80% of cases with slow response treated with a HR trial achieved CR. For these patients allogeneic HSCT was performed according to physicians’ criteria, resulting in only 5/18 CR patients (including the two patients who had an excess of MRD at the end of the first induction) being transplanted, a proportion that is clearly lower than that expected for ALL patients presenting chemoresistance. As expected for HR ALL, one third of the patients relapsed.^7^ It could be speculated that the proportion of relapses would have been lower if all patients had undergone allogeneic HSCT, as the frequency of relapses was lower in transplanted than in nontransplanted patients (1/5 for allogeneic HSCT vs 5/13 for chemotherapy), but the low number of cases in this series does not allow to draw definitive conclusions. Despite the OS for these patients was lower (and the CIR slightly higher) than that observed for patients showing good early response, the differences did not reach statistical significance, indicating that a substantial proportion of these patients can be successfully rescued if they are transferred early to a HR protocols. However, the absence of statistically significant differences in OS between patients on trial on those who were rescued in HR trials does not mean that the rescue therapy was really very effective (40% of relapses). It is clear that other approaches are necessary to improve the prognosis of this subset of patients, as discussed later in this section.

Although the OS rates observed in the ALLRE08 and the ALL96 trials were equivalent, some differences should be pointed out. Thus, while no differences existed in the inclusion criteria and the baseline characteristics of the patients enrolled in both trials, significantly lower OS rates were observed in the here reported trial for YA vs adolescents, not being observed in the ALL96 trial, where the two groups of patients showed similar outcome.^12^ This might be due to a better OS of adolescents in the ALLRE08 trial vs the ALL96 study (87% [74%‐100%] vs 80% [67%‐93%]) and a lower OS for YA (as a consequence of a higher CIR) in this vs the previous trial (63% [46%‐80%] vs 71% [58%‐84%]) emphasizing in the ALLRE08 trial the overall difference in OS observed between adolescents and YA, that was already present at a nonsignificant level in the ALL96 trial.^13^

Overall, the tolerability of the ALLRE08 trial was good, probably due to the relatively conservative upper age limit for inclusion, set at 30 years. Main toxic events were due to cytopenias and infections, followed by liver toxicity and hypersensitivity to *E coli* asparaginase. Although the frequency of delays and dose modification of the chemotherapeutic drugs were high, especially during maintenance, there were no differences between adolescents and YA, and these modifications did not show impact on patients’ outcome. Dose modifications and delays are important in the pediatric setting, but their relevance in AYA and adults has been less studied.

Despite the long follow‐up of the patients here reported, some limitations should be pointed out for this trial. First, the definition of risk factors did not take into account important features such as deep molecular characterization of the patients at baseline aimed to detect specific gene deletions/mutations, as well as other important characteristics with recognized prognostic impact such as the *BCR‐ABL* like signature or the early pre‐T signature, that are frequent among AYA,^22^, ^23^, ^24^ although the latter subgroups were identified when the trial was in an advanced phase of recruitment. It is clear that at present the risk definition should include the aforementioned studies. Second, combined cytological and MRD criteria were used to identify poor early responders, but only two patients were transferred to a HR protocol because of poor MRD clearance. Despite the early cytological response has been extensively used in the past for early identification of this subset of patients resistant to induction therapy, and has prognostic significance,^25^ most modern trials use end‐induction MRD as the only tool to assess the quality of response, given that there is not a precise correlation between poor early cytological response and the pattern of MRD clearance.^11^ In turn, the end‐induction MRD cut‐off (0.25%) selected for transferring the patients to a HR trial can be considered too high, since most modern trials consider lower MRD cut‐offs (>0.1% or even > 0.01%) to define poor MRD response after induction. Third, MRD was not centrally assessed, and consequently introduced a potential bias in the study, potential impact on the reproducibility of the results. In addition, the MRD was assessed by 4‐color FCM, a technique known now to provide insufficient sensitivity. Finally, the study was restricted to AYA with SR features, which might limit direct comparison with other trials that include AYA with Ph‐negative ALL, only selected by age.

At present, there is room for improvement of patients outcome after pediatric type therapy in AYA with Ph‐neg ALL, and according to our results, this improvement is especially necessary for YA.^26^ The more extensive use of conventional drugs such as PEG‐asparaginase^11^, ^17^ together with the early incorporation of immunotherapeutic approaches with naked (anti CD20),^27^ immunoconjugated (inotuzumab ozogamicin),^28^ or bispecific (blinatumomab)^29^, ^30^ monoclonal antibodies which have proven effective in relapsed/refractory or MRD positive patients might contribute to improve the quality of remission and decreased the relapse rate observed in our study. In addition, early use of genetically engineered T cells that recognize ALL epitopes (CAR T cells) will also provide an opportunity to improve the outcomes of these patients.^31^, ^32^, ^33^ Finally, incorporation of targeted kinase inhibitors to pediatric type chemotherapeutic regimens for specific subtypes of ALL such as *BCR‐ABL* like might further contribute to overcome the resistance to therapy and improve survival of this particularly difficult to treat subgroup of ALL patients.

## CONFLICT OF INTEREST

The authors made no disclosures.

## AUTHOR CONTRIBUTIONS

JMR was primarily responsible for the design of the trial and wrote the paper. MM analyzed the data. The remaining authors (PM, MT, DMC, JGC, CG, PB, RGB, RC, MP, JR, SM, SV, AN, EG, JMHR, JB, MLA, FV, PMS, MC, IGC, AGG, MJSS, MC, EF, and AO) qualified for authorship according to the WAME criteria, reported the patients and followed them clinically. These contributions explain the order of authors.

## Data Availability

The data that support the findings of this study are available from the corresponding author upon reasonable request.
