# Supplementary material for: A pediatric regimen for adolescents and young adults with Philadelphia chromosome‐negative acute lymphoblastic leukemia: Results of the ALLRE08 PETHEMA trial
Source: Cancer Med. 2020 Feb 5;9(7):2317–29. doi: 10.1002/cam4.2814 (PMC7131850; doi:10.1002/cam4.2814)
Supplement: Supplementary file 1 [file CAM4-9-2317-s001.docx]

Supplemental Table 1. Treatment regimens of high-risk trials.

| Phase | Week No. | HR-ALL03 trial | HR-ALL11 trial |
| --- | --- | --- | --- |
| Induction-1 | | | |
| Vincristine | 1-4 | 1.5mg/m2 (max. 2) days 1,8,15,22 | 1.5mg/m2 (max. 2) days 1,8,15,22 |
| Daunorubicin | 1-4 | 60mg/m2 days 1,8,15,22 | 45mg/m2 days 1,8,15,22 |
| Prednisone | 1-4 | 60mg/m2 days 1-27 | 60mg/m2 days 1-14  20mg/m2 days 15-21  15mg/m2 days 21-28 |
| Mitoxantrone^1^ | 3 | 12mg/m2 x3 | - |
| Cytarabine^1^ | 3 | 2g/m2 bid x2 | - |
| Asparaginase | 3 | - | PEG 2,000 UI/m2 day 15 or L-ASP 10,000UI/m2 16-20 and 23-27 |
| Induction-2^2^ | | | |
| Fludarabine | 5 | - | 30mg/m2 days 1-5 |
| Cytarabine | 5 | - | 2g/m2 days 1-5 |
| Idarubicin | 5 | - | 12mg/m2 days 1,3,5 |
| Early consolidation 1 | | | |
| Vincristine | 7-8 | 1.5mg/m2 (max. 2) days 1,8 | 1.5mg/m2 (max. 2) days 1,8 |
| Dexamethasone | 7-8 | 20mg/m2 days 1-5  10mg/m2 day 6  5mg/m2 day 7  2.5 mg/m2 day 8 | 20mg/m2 days 1-5  10mg/m2 day 6  5mg/m2 day 7  2.5 mg/m2 day 8 |
| Methotrexate | 7 | 3g/m2 day 1 | 5g/m2 day 1 |
| Cytarabine | 7 | 2g/m2 bid day 5 |  |
| Asparaginase | 7 | E.coli ASP 25,000 IU/m2 day 5 | PEG 2000 UI/m2 day 3 or E.coli ASP 20,000 IU/m2 day 3 |
| Mercaptopurine | 7 | 100mg/m2 days 1-5 | - |
| Early consolidation 2 | | | |
| Vincristine | 11-12 | 1.5mg/m2 (max. 2) days 1,8 | - |
| Dexamethasone | 11-12 | 20mg/m2 days 1-5  10mg/m2 day 6  5mg/m2 day 7  2.5 mg/m2 day 8 | 20mg/m2 days 1-5  10mg/m2 day 6  5mg/m2 day 7  2.5 mg/m2 day 8 |
| Methotrexate | 11 | 3g/m2 day 1 | - |
| Cyclophosphamide | 11 | 150mg/m2 days 1-5 | - |
| L-asparaginase | 11 | 25,000 IU/m2 day 5 | PEG 2,000 UI/m2 day 3 or E.coli ASP 20,000 IU/m2 day 3 |
| Mitoxantrone | 11 | 12mg/m2 day 5 | - |
| Cytarabine | 11 | - | 2g/m2 bid days 1,2 |
| Early consolidation 3 |  | | Repeats Early consolidation 1 |
| Dexamethasone | 15-16 | 20mg/m2 days 1-5  10mg/m2 day 6  5mg/m2 day 7  2.5 mg/m2 day 8 | - |
| Cytarabine | 15 | 2g/m2 bid days 1,2 | - |
| Teniposide | 15 | 150mg/m2 days 3,4 | - |
| L-Asparaginase | 15 | 25,000 IU/m2 day 5 | - |
| Late consolidation 1^3^ | 19-20 | Repeats Early consolidation 1 | Repeats Early consolidation 1 |
| Late consolidation 2^3^ | 23-24 | Repeats Early consolidation 2 | Repeats Early consolidation 2 |
| Late consolidation 3^3^ | 27-28 | Repeats Early consolidation 3 | Repeats Early consolidation 3 |

ASP: asparaginase; PEG ASP: pegylated asparaginaseSupplemental Table 2. Comparison of the main outcomes for the ALLRE08 and ALL96 PETHEMA protocols for the whole patient series and the adolescent and young adults groups.

| **Patient group** | **ALL96 trial**  **(n=84)** | **ALLRE08 trial**  **(n=89)** | **P value** |
| --- | --- | --- | --- |
| Slow responders, n (%) | 9/83 (11%) | 21 (24%) | 0.028 |
| CR rate, n (%) | 82 (98%) | 84/88 (95%) | 0.682 |
| 5-yr. OS (95%CI) | 75% (65% ; 85%) | 74% (63% ; 85%) | 0.969 |
| 5-yr. CIR (95%CI) | 31% (21% ; 42%) | 35% (23% ; 47%) | 0.182 |
| 5-yr. OS (95%CI) for adolescents | 80% (67% ; 93%) | 87% (74% ; 100%) | 0.341 |
| 5-yr. OS (95%CI) for YA | 71% (58% ; 84%) | 63% (46% ; 80%) | 0.424 |
| 5-yr. CIR (95%CI) for adolescents | 34% (18% ; 50%) | 13% (4% ; 28%) | 0.515 |
| 5-yr. CIR (95%CI) for YA | 38% (21% ; 54%) | 52% (34% ; 67%) | 0.024 |

CR: complete response; OS: overall survival; CI: confidence interval; YA: young adults
